# Health sciences library outreach to family caregivers: a call to service

**DOI:** 10.5195/jmla.2018.390

**Published:** 2018-04-01

**Authors:** Mary M. Howrey

**Affiliations:** Executive Director/Medical Librarian, TLM Network Special Library, Member Library of the National Network of Libraries of Medicine Southeastern/Atlantic Region, Transforming Lives & More Network, 9906 NW 76th Street, Tamarac, FL 33321

## Abstract

This commentary discusses the information needs of family caregivers and care recipients in the United States. Health sciences library services and outreach activities that support family caregivers include: (1) advocacy, (2) resource building, and (3) programming and education. Ethical issues related to the privacy and confidentiality of clients are outlined in the commentary for information service providers. Also, continuing professional education resources are identified to assist librarians in providing high-quality information services for this special family caregiver population, such as those designed by the National Library of Medicine (NLM) through the NLM 4 Caregivers program.

## FAMILY CAREGIVERS: A TARGET AUDIENCE FOR HEALTH SCIENCES LIBRARIES

Supporting the health information needs of individuals and families, including underserved special populations, is one of the primary competencies of health sciences librarians identified in the 2017 Medical Library Association (MLA) competency and professional success guidelines [1]. One large but underserved segment of the US population, family caregivers and their care recipients, is presently off the radar and is mostly an invisible audience for health sciences libraries. These individuals are an innovative and receptive target audience for library program planners and managers who hope to have an impact on health information literacy and the quality of life for families.

A recent report from the Bureau of Labor Statistics, *Unpaid Eldercare in the United States,* points to the large number of US family caregivers. Of the total US civilian population in 2015–2016, 41.3 million people over 14 years of age (16%) provided unpaid eldercare [2]. In particular, there are a growing number of individuals and families in the caregiving population affected by Alzheimer’s disease. Five million persons are currently afflicted with Alzheimer’s disease, and the Alzheimer’s Association projects 16 million cases of Alzheimer’s disease by 2050 [3]. Assessing family caregiver information needs at the point of need is important because the progression of Alzheimer’s disease is often unclear to families, and there is much frustration with health care providers since there is no effective disease prevention or medical treatment for Alzheimer’s disease symptoms. Given poor treatment outcomes, Alzheimer’s patients are more likely to suffer from behavioral issues that are challenging and burdensome for caregivers compared with other chronic diseases, as reported in a 2015 National Alliance for Caregiving (NAC) and AARP family caregiver study [4].

Family caregivers provide informal and unpaid support to adults who are patients suffering from intellectual or developmental disabilities, chronic diseases, or cognitive impairment. At the same time that caregivers are assisting a spouse, parent, sibling, or special needs adult with activities of daily living; providing nursing care; and serving as “information proxies” [5], they also need to be taking care of themselves and may be raising their own children. Thus, the popular media depicts family caregivers as part of the “sandwich generation,” who experience high levels of stress, feelings of burden, depression, and poor health outcomes due to juggling work with home responsibilities [6].

In contrast to the challenges and negative media images associated with caregiving, current research shows that family caregivers receive many positive benefits from their unpaid caregiving responsibilities. As Brown and Brown [7] and Anderson and White [8] reported, caregivers experience a sense of satisfaction, purpose, and well-being plus longevity gained from their compassionate interactions with care recipients and the social support received from others. The 2017 AARP survey of 1,091 family caregivers supports these research findings, showing that positive emotions outweigh negative emotions for 91% of family caregivers in the representative sample, and 54% of caregivers experienced unexpected joy in caregiving [9].

Direct services to family caregivers are generally seen to be the domain of social workers, health care professionals, and government agencies such as county area agencies on aging or state departments of public aid. Yet, health sciences librarians have an opportunity for important outreach to family caregivers by collaborating with other health care professionals and community agencies. Innovative medical library partnerships with community agencies that directly serve family caregivers and care recipients establish libraries as change agents and enable librarians to transform the lives of family caregivers and their loved ones through advocacy, education, public programming, and resource-building of easily accessible in-house library and online resources.

## THE ECONOMICS OF FAMILY CAREGIVING IN THE UNITED STATES

As NAC and AARP reported in 2015, the economic value of informal caregiving services provided by over 40 million US family caregivers was $470 billion annually, with 40% of caregivers unemployed and faced with financial shortfalls [10]. Levy, an attorney and gerontologist, writes in his practical guide for caregivers that, over a lifetime, all individuals will provide care to a family member, friend, or neighbor and will likely require care for themselves [11]. As a consequence of high medical care expenses later in life, Levy advises that advance planning is essential for everyone.

The Centers for Disease and Control and Prevention report that family caregivers will be in short supply by 2030, with only four potential family caregivers per adult, as compared to seven potential caregivers in 2016. This caregiving shortage is due to the doubling of the number of people aged sixty-five and older between 2000 and 2030. By 2030, all baby boomers will be at least sixty-five years or older, totaling seventy-one million Americans [12]. Based on the sheer size of the US care recipient population, it is clear that informal family caregivers serve today and in the future as the “backbone” of the US long-term care system. Family caregivers and their care recipients are a strong and novel target audience with unique information needs for health sciences library outreach.

## THREE RECOMMENDED STRATEGIES FOR HEALTH SCIENCES LIBRARY OUTREACH SERVICES TO FAMILY CAREGIVERS

What can health sciences librarians, who are employed in a variety of work organizations, do to establish effective library services and programs to support the health information literacy, resourcefulness, and resilience of the diverse family caregivers? This commentary offers recommendations, strategies, and a call to service for health sciences library decision-makers and librarians who plan and implement community outreach services and establish health promotion partnerships with local community agencies, government officials, and employers. Three strategies are recommended for developing health sciences library outreach services to family caregivers.

### Strategy #1: Advocacy for caregiving public policy

Health sciences libraries can offer public programs that introduce current legislation, public policy, and budget priorities at the federal, state, and local levels that support basic resources for diverse family caregivers and their families. Speakers can be invited from local organizations such as AARP, the League of Women Voters, the Urban League, area agencies on aging, or city, county, and state governments to discuss topics such as the following.

- Introduced on July 27, 2017, by Representatives Michelle Lujan Grisham (NM) and Ileana Ros-Lehtinen (FL), the Care Corps Demonstration Act of 2017 will place volunteers in communities to work with seniors and individuals with disabilities who need extra support to live independently. In exchange, volunteers will receive health insurance and other benefits, such as tuition assistance [13].
- The RAISE Family Caregivers Act of 2017 House bill was passed by the Senate on January 9, 2018, and became public law no. 115-119 when signed by President Trump on January 22. This law authorizes the secretary of the Department of Health and Human Services to develop a national strategy to support family caregivers. An advisory agency with representatives from the public and private sectors will be formed to make recommendations and to identify specific actions for government, local communities, employers, and service providers. The intention is to support and recognize family caregivers with recommendations and specific activities updated on an annual basis [14].
- The Caring for Credit Act of 2017 was introduced May 17, 2017, by Representatives Tom Reed II (NY) and Linda Sanchez (CA). The bill amends the Internal Revenue Code to allow an eligible caregiver a new tax credit for 30% of the cost of long-term care expenses that exceed $2,000, up to $3,000 in a taxable year. The bill defines “eligible caregiver” as an individual who has earned income for the taxable year in excess of $7,500 and pays or incurs expenses for providing care to a spouse or other dependent relative with long-term care needs [15].
- Funding for the Medicaid Program (US Centers for Medicare and Medicaid Services) is provided by the states and US federal government for health services to low-income pregnant women and children, the elderly, and disabled individuals [16]. Medicaid covers sixty-nine million persons as of May 2017 and supports six of every ten nursing home residents according to the Henry J. Kaiser Family Foundation [17].
- The Family Medical Leave Act (FMLA) entitles eligible employees of covered employers (with fifty or more employees within seventy-miles miles of a work location) to take unpaid, job-protected leave for specified family and medical reasons and guarantees continuation of group health insurance coverage under the same terms and conditions as if the employee had not taken leave [18].

### Strategy #2: Resource-building

Health sciences libraries can target family caregivers with high-quality resources by:

- developing a web page or LibGuide for caregivers with print and online resources available in their libraries;
- creating a web page to easily navigate local community resources with services targeted to caregivers of seniors and people with disabilities, such as those found on the Transforming Lives and More Network Special Library’s website [19];
- highlighting National Library of Medicine (NLM) health information resources using the NLM 4 Caregivers toolkit [20];
- referring patrons to the National Caregivers Library website with online articles, reports, forms, checklists, and website links [21]; and
- consulting the National Rehabilitation Information Center (NARIC) website for a portable document format (PDF) copy of “Information Resources for Caregiving & Caregivers: Quick Picks from Librarians at NARIC” to share with family caregivers as well as health care and social service professionals [22].

### Strategy #3: Programming and education

Health sciences librarians can offer outreach programs to support and celebrate the contributions of family caregivers. Excellent examples include:

- programs for National Family Caregivers Month in November each year with programming tips from Gale Cengage [23] and the Caregiver Action Network [24];
- information literacy training programs for family caregiver support groups that are developed by medical librarians [25];
- health education and “self-help” resource fairs similar to the Thriving Families=Healthier Communities program in Broward County, Florida, that highlighted health insurance programs such as Medicare and Medicaid, community services available for older adults and persons with disabilities, legal and financial matters, respite and adult day care, palliative and hospice care, caregiver support groups, and personal care and empowerment [26];
- local community educational programs such as AARP TEK Careversations workshops [27] and Today’s Caregiver Fearless Caregiver Conferences [28]
- the Caregiver Library Project of Allegheny County’s Human Services/Area Agency on Aging, a pilot program in Pennsylvania initiated with five public libraries that quickly grew into an outreach program of fifty public libraries, offering backpacks containing a variety of information resources for caregivers to check out and take home to review [29]; and
- the Christina Healthcare System (Wilmington, DE) Medical Libraries’ “Healing through Creativity” education program for cancer patients and their families [30].

## ETHICAL CONSIDERATIONS IN SERVING FAMILY CAREGIVERS

Both the MLA and American Library Association (ALA) codes of ethics stress that information professionals are responsive and respect the privacy and confidentiality of caregivers and their care recipients. It is especially important that health sciences librarians work collaboratively with physicians and social services professionals at the point of need to supply high-quality and appropriate clinical and consumer health information for family caregivers that reflect patient preferences, needs, literacy levels, and cultural values. Health sciences librarians must deliver health information services that are consistent with Health Insurance Portability and Accountability Act (HIPAA) requirements and design programs that are in line with the patient-centered care model introduced by the Institute of Medicine [31]. Accessible and high-quality health information services incorporate these eight family-centered care principles: (1) respect, (2) coordination and integration of care, (3) information and education, (4) physical comfort, (5) emotional support, (6) involvement of family and friends, (7) continuity and transition, and (8) access to care [32].

Health sciences librarians, in supplying health information and education and offering referrals to local community agencies and services, contribute to a patient care plan that eases the transitions, frustrations, and stress that family caregivers and their care recipients experience. As members of the family-centered health care team, librarians empower family caregivers with information for informed and autonomous decision-making. Health sciences library information services and family-centered care values contribute to continuity of care, caregiver well-being, patient comfort, and a high quality of life for patients and their families.

## CONTINUING PROFESSIONAL EDUCATION ABOUT FAMILY CAREGIVERS AND FUTURE US TRENDS

It is important that health sciences librarians pursue continuing professional education to expand their professional knowledge of family caregivers and their information needs. Continuing education opportunities include both formal and informal self-directed learning such as: (1) assessing the information needs of caregivers and older adults, (2) conducting or retrieving local community surveys, (3) studying family caregiver education websites and attending caregiver workshops or conferences, and (4) reviewing the library and health sciences literature and general news sources for best practices.

Results of the 2015 family caregiver survey conducted by NAC and AARP pointed to the information needs of family caregivers and the typical problems that they face [4]. By reading the NAC and AARP report, *Caregiving in the U.S.,* librarians will easily discover the types of information that caregivers seek. For example, among the 1,087 unpaid family caregivers surveyed, NAC and AARP found that more than 8 out of 10 caregivers (84%) who were providing care to someone 50 years of age or older said that they could use more information or help with caregiving topics in general. Family caregivers most often wanted information about keeping their loved ones safe at home (43%) and managing their own stress (42%). About 1 in 4 caregivers (24%) who were providing care to someone aged 50 and older would like more information about making end-of-life decisions and hospice care. Managing challenging and difficult behaviors was reported as an information need by 16% of caregivers, while a need for information about managing incontinence or toileting problems was indicated by 11% of caregivers.

A local community needs analysis using available public health data sources is an important next step for librarian professional development because the future number of potential family caregivers is staggering, and community-level planning is critical for health sciences library program development [33]. Qualitative research methods such as action research and interviews require that librarians assess needs interactively with family caregiver focus groups, family support groups, regional caregiver and aging coalitions, business advisory councils, public library boards, government agencies serving older adults, and churches. Networking with community groups accelerates librarians’ learning, stimulates innovation, and offers practical insights into service gaps and community resources that influence the quality of care for caregivers and their care recipients.

Studying family caregiver websites can aid in developing and implementing library-based family caregiving education programs. The premiere family caregiver education curriculum is offered by the Rosalynn Carter Institute for Caregiving (RCIC) [34]. This evidence-based workshop curriculum is continuously revised and is now in its third edition. It focuses on caregiver identity, problem-solving skills, and online community resource navigation and retrieval. Additional websites for continuing education, research, and review are the NLM 4 Caregivers toolkit [20] and the US Administration on Aging’s Eldercare Locator database of community services [35].

Health sciences information professionals can attend local support group meetings and online conferences sponsored by self-help organizations such as AARP, the Alzheimer’s Association, the American Cancer Society, the American Heart Association, the American Diabetes Association, Caregiving.com, NAC, and Today’s Caregiver. Attendance at support group meetings and online conferences builds both knowledge and empathy. More specifically, AARP and the Alzheimer’s Association offer cost-effective training opportunities for librarians. AARP offers Careversations workshops [27] and the Alzheimer’s Association sponsors family support groups, annual educational conferences, and online family caregiver certification [36].

Finally, a review of the professional literature must include PubMed Health [37] to identify evidence-based sources to answer caregiver clinical questions about nursing and medical care. According to Levy, clinical questions constitute 15% of the information that caregivers need for their care recipients [11]. General search engines, such as Google and Bing, and licensed scholarly databases, such as Library Literature & Information Science Full Text [38], complement each other in identifying model library outreach programs for family caregivers.

A complete family caregiving literature review should include the “landmark” report, *Families Caring for an Aging America,* which analyzes demographics, national policies, health care system improvements, and evidence-based programs projected into the future through 2050 to support families [39]. The Pew Research Center issues reports periodically about caregiving issues and national trends that impact families [40]. Furthermore, the Family Caregiver Alliance website provides state-specific caregiving statistics [41] and useful family caregiving online resources [42]. To stay current and connected to high-quality and culturally diverse family caregiving resources for information and referral purposes, health sciences librarians can register for free alerting services and caregiver communities that are offered by select national organizations (Table 1).

**Table 1:**
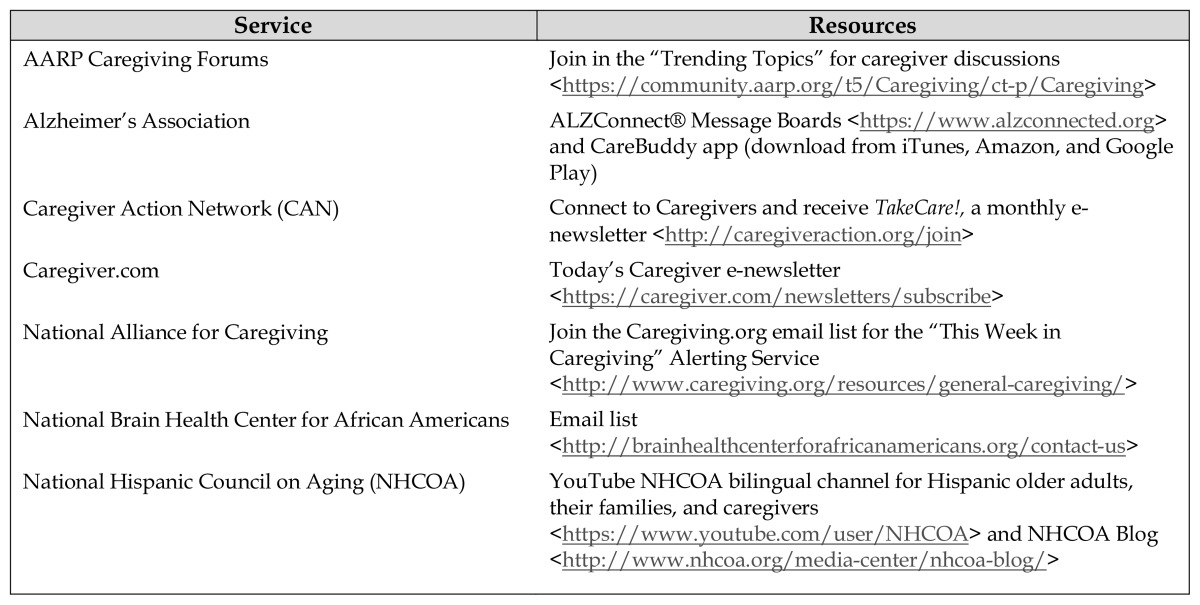
Current awareness services from select family caregiving associations

Service	Resources
AARP Caregiving Forums	Join in the “Trending Topics” for caregiver discussions <https://community.aarp.org/t5/Caregiving/ct-p/Caregiving>
Alzheimer’s Association	ALZConnect® Message Boards <https://www.alzconnected.org> and CareBuddy app (download from iTunes, Amazon, and Google Play)
Caregiver Action Network (CAN)	Connect to Caregivers and receive *TakeCare!,* a monthly e-newsletter <http://caregiveraction.org/join>
Caregiver.com	Today’s Caregiver e-newsletter <https://caregiver.com/newsletters/subscribe>
National Alliance for Caregiving	Join the Caregiving.org email list for the “This Week in Caregiving” Alerting Service <http://www.caregiving.org/resources/general-caregiving/>
National Brain Health Center for African Americans	Email list <http://brainhealthcenterforafricanamericans.org/contact-us>
National Hispanic Council on Aging (NHCOA)	YouTube NHCOA bilingual channel for Hispanic older adults, their families, and caregivers <https://www.youtube.com/user/NHCOA> and NHCOA Blog <http://www.nhcoa.org/media-center/nhcoa-blog/>

## A CALL TO SERVICE TO SUPPORT FAMILY CAREGIVERS

This commentary is a call to service for health sciences librarians to provide outreach to family caregivers and their care recipients. This library outreach mission reinforces our professional service role as empathetic and mindful leaders in our workplaces and local communities. Strategies outlined include advocacy for caregiving legislation and public policy, library resource-building, partnership development with public libraries and community agencies, and strengths-based family caregiver training. These cutting-edge library best practices represent the trail-blazing capacity, responsiveness, and innovativeness of health sciences librarians.

Receiving emotional support and authoritative information from librarians, who are part of the health care team, uplifts and unleashes the resourcefulness, resilience, and strengths of family caregivers. As proactive members of the health care team, librarians can embrace and honor family caregivers and their care recipients through expanded library outreach and information services. The recommendations for health sciences library outreach services build family caregiver strengths. As discussed by Bailey and Gordon, family empowerment, personhood, problem-solving, and a positive outlook are beneficial outcomes for caregivers, who are fully accepted as part of the patient’s care team and are offered unconditional support and acknowledgment [43].

With our proactive and mindful professional commitment to family caregivers, health sciences librarians can creatively address caregiving challenges, introduce a wide range of practical solutions to address problems, focus mindfully on the present moment, support the establishment of a broad “circle of care” and social safety network, and advance the vision of a better world filled with hope and compassion for family caregivers, their loved ones, their health care teams, our local communities, and ourselves as librarians [44].
